# The genome sequence of the Common Sheetweb Spider *Linyphia triangularis* (Clerck, 1757)

**DOI:** 10.12688/wellcomeopenres.23754.1

**Published:** 2025-02-24

**Authors:** Olga Sivell, Duncan Sivell, Natural History Museum Genome Acquisition Lab, Darwin Tree of Life Barcoding collective, Wellcome Sanger Institute Tree of Life Management, Samples and Laboratory team, Wellcome Sanger Institute Scientific Operations: Sequencing Operations, Wellcome Sanger Institute Tree of Life Core Informatics team, Tree of Life Core Informatics collective, Darwin Tree of Life Consortium

**Affiliations:** 1 Natural History Museum, London, England, UK

**Keywords:** Linyphia triangularis, Common Sheetweb Spider, genome sequence, chromosomal, Araneae

## Abstract

We present a genome assembly from a male *Linyphia triangularis* (Common Sheetweb Spider Arthropoda; Arachnida; Araneae; Linyphiidae). The genome sequence has a total length of 1,349.10 megabases. Most of the assembly (95.36%) is scaffolded into 13 chromosomal pseudomolecules, including the X _1_ and X _2_ sex chromosomes. The mitochondrial genome has also been assembled and is 15.31 kilobases in length.

## Species taxonomy

Eukaryota; Opisthokonta; Metazoa; Eumetazoa; Bilateria; Protostomia; Ecdysozoa; Panarthropoda; Arthropoda; Chelicerata; Arachnida; Araneae; Araneomorphae; Entelegynae; Orbiculariae; Araneoidea; Linyphiidae; Linyphiinae; Linyphia; *Linyphia triangularis* (Clerck, 1757) (NCBI:txid94031)

## Background

*Linyphia triangularis* (Clerck, 1757) is a species belonging to the family Linyphiidae (Araneae), commonly known as money spiders. It is the largest spider family in Britain with approximately 280 species in 123 genera ( Bee *et al.*, 2017). *L. triangularis* is fairly large for a linyphiid, males measuring 4.6–6.0 mm and females 5.0–6.6 mm. This species is easy to recognise by the pale brown carapace with dark brown margins and a “tuning fork” medial mark, yellowish brown legs with numerous spines and femora without dark spots. The female has a purplish abdominal pattern with pale, creamy-white and black streaks on the sides. The drawings of epigyne and chelicerae are provided by Roberts (1996).

*Linyphia triangularis* is a ubiquitous, common and widely distributed Palaearctic species that is abundant and widespread in Britain ( Bee *et al.*, 2017; GBIF Secretariat, 2024; Harvey *et al.*, 2002; Roberts, 1996). It has been accidentally introduced to North America. It became established in Maine (USA) where it outcompetes the native linyphiid species *Frontinella communis* (Hentz, 1850) ( Jennings *et al.*, 2002; Simpson *et al.*, 2023) *.* The more aggressive European species likely contributes to the decline of *F. communis* by taking over their webs (and perhaps consuming the original occupier) or incorporating them into its own webs. Web take-over or incorporation is a typical behaviour of *L. triangularis*. Moreover, *F. communis* avoids areas inhabited by *L. triangularis* and abandons its webs when *L. triangularis* is introduced on its territory ( Bednarski *et al.*, 2010; Houser *et al.*, 2014).

*Linyphia triangularis* builds sheet web in segments over many days. This consists of a supporting structure and a meshwork of fibres of varying thickness ( Benjamin *et al.*, 2002; Benjamin & Zschokke, 2004). The web is typically suspended among vegetation, up to six metres above ground and the spider resides on the underside awaiting prey ( Bee *et al.*, 2017).

*Linyphia triangularis* has an annual life cycle. It reaches maturity in July to August and adults occur until September to October. The males mature a few days earlier and leave their webs in search of females. Near-mature females can be guarded by males for several days and copulation occurs immediately after female’s final moult. If more than one male is present on the web, they fight till one of them retreats or dies and the remining male copulates with the female. The copulation can also be interrupted by the arrival of another male or other disturbance in which case a female will retreat to the edge of the web. She can mate for a second time and potentially produce offspring from two males. Detailed descriptions of courtship and copulation of *L. triangularis* was provided by Rovner (1968), Toft (1989), Nielsen and Toft (1990), Stumpf (1990), Schulz and Toft (1993), Weldingh *et al.* (2011). The eggs are laid in October, into a sac in which they overwinter ( Stumpf, 1990).

The phylogeny of Linyphyiidae has been researched in number of studies using morphological and/or molecular data ( Arnedo *et al.*, 2009; Gavish-Regev *et al.*, 2013; Hormiga, 2000; Hormiga & Scharff, 2005; Miller & Hormiga, 2004; Tu & Hormiga, 2011; Wang *et al.*, 2015). The genus *Linyphia* Latreille, 1804 is monophyletic according to Arnedo *et al.* (2009). The family was recovered as monophyletic by Arnedo *et al.* (2009), but not by Wang *et al*. (2015). Within Linyphiidae seven well supported clades, inconsistent with the seven currently recognized subfamilies, and four unstable branches have been recovered by Wang *et al*. (2015). After removing the unstable branches, the remining Linyphiidae were recovered as monophyletic ( Wang *et al.*, 2015). The further work is needed to resolve phylogenetic relationships within Linyphyiidae.

The high-quality genome of *L. triangularis* presented here will aid research into the phylogeny and taxonomy of the Linyphyiidae. It was sequenced from specimen collected on 29/07/2021 from Hartslock Nature Reserve, England by Duncan and Olga Sivell and identified by Olga Sivell following Bee *et al.* (2017) and Roberts (1996). The genome was sequenced as part of the Darwin Tree of Life Project, a collaborative effort to sequence all named eukaryotic species in the Atlantic Archipelago of Britain and Ireland.

## Genome sequence report

### Sequencing data

**Figure 1.**
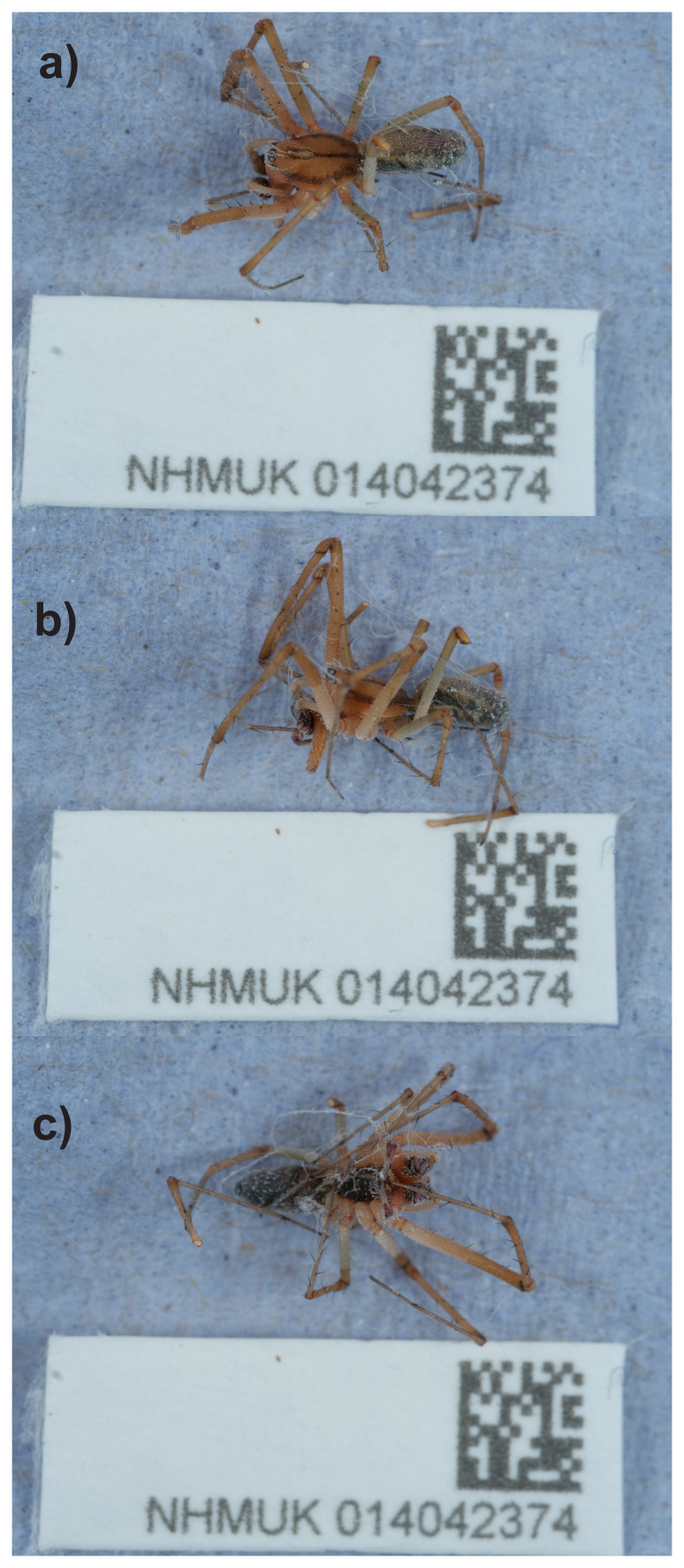
Photograph of the *Linyphia triangularis* (qqLinTria7) specimen used for genome sequencing.

The genome of a specimen of *Linyphia triangularis* ( Figure 1) was sequenced using Pacific Biosciences single-molecule HiFi long reads, generating 82.72 Gb from 87.32 million reads. GenomeScope analysis of the PacBio HiFi data estimated the haploid genome size at 1,258.44 Mb, with a heterozygosity of 1.13% and repeat content of 37.68%. These values provide an initial assessment of genome complexity and the challenges anticipated during assembly. Based on this estimated genome size, the sequencing data provided approximately 63.0x coverage of the genome. Chromosome conformation Hi-C sequencing produced 104.61 Gb from 692.76 million reads. Table 1 summarises the specimen and sequencing information.

**Table 1.** Specimen and sequencing data for *Linyphia triangularis*.

Project information			
**Study title**	Linyphia triangularis (European sheetweb spider)		
**Umbrella BioProject**	PRJEB65733		
**Species**	*Linyphia triangularis*		
**BioSpecimen**	SAMEA111458582		
**NCBI taxonomy ID**	94031		
Specimen information			
**Technology**	**ToLID**	**BioSample accession**	**Organism part**
**PacBio long read sequencing**	qqLinTria7	SAMEA111458661	head and thorax
**Hi-C sequencing**	qqLinTria9	SAMEA111458698	head and thorax
Sequencing information			
**Platform**	**Run accession**	**Read count**	**Base count (Gb)**
**Hi-C Illumina NovaSeq 6000**	ERR12035316	6.93e+08	104.61
**PacBio Revio**	ERR12015774	7.32e+06	82.72

### Assembly statistics

The primary haplotype was assembled, and contigs corresponding to an alternate haplotype were also deposited in INSDC databases. The assembly was improved by manual curation, which corrected 47 misjoins or missing joins and removed 6 haplotypic duplications. These interventions reduced the total assembly length by 2.23%, decreased the scaffold count by 5.08%, and decreased the scaffold N50 by 0.52%. The final assembly has a total length of 1,349.10 Mb in 373 scaffolds, with 289 gaps, and a scaffold N50 of 96.46 Mb ( Table 2).

**Table 2.** Genome assembly data for *Linyphia triangularis*.

Genome assembly		
Assembly name	qqLinTria7.1	
Assembly accession	GCA_963978545.1	
*Alternate haplotype accession*	*GCA_963978535.1*	
Assembly level for primary assembly	chromosome	
Span (Mb)	1,349.10	
Number of contigs	662	
Number of scaffolds	373	
Longest scaffold (Mb)	127.92	
Assembly metric	Measure	*Benchmark*
Contig N50 length	6.25 Mb	*≥ 1 Mb*
Scaffold N50 length	96.46 Mb	*= chromosome N50*
Consensus quality (QV)	Primary: 59.5; alternate: 59.7; combined 59.6	*≥ 40*
*k*-mer completeness	Primary: 82.97%; alternate: 70.52%; combined: 99.10%	*≥ 95%*
BUSCO *	C:96.0%[S:90.8%,D:5.2%], F:0.8%,M:3.1%,n:2,934	*S > 90%; D < 5%*
Percentage of assembly mapped to chromosomes	95.28%	*≥ 90%*
Sex chromosomes	X _1_ and X _2_	*localised homologous pairs*
Organelles	Mitochondrial genome: 15.31 kb	*complete single alleles*

* BUSCO scores based on the arachnida_odb10 BUSCO set using version 5.5.0. C = complete [S = single copy, D = duplicated], F = fragmented, M = missing, n = number of orthologues in comparison.

**Figure 2.**
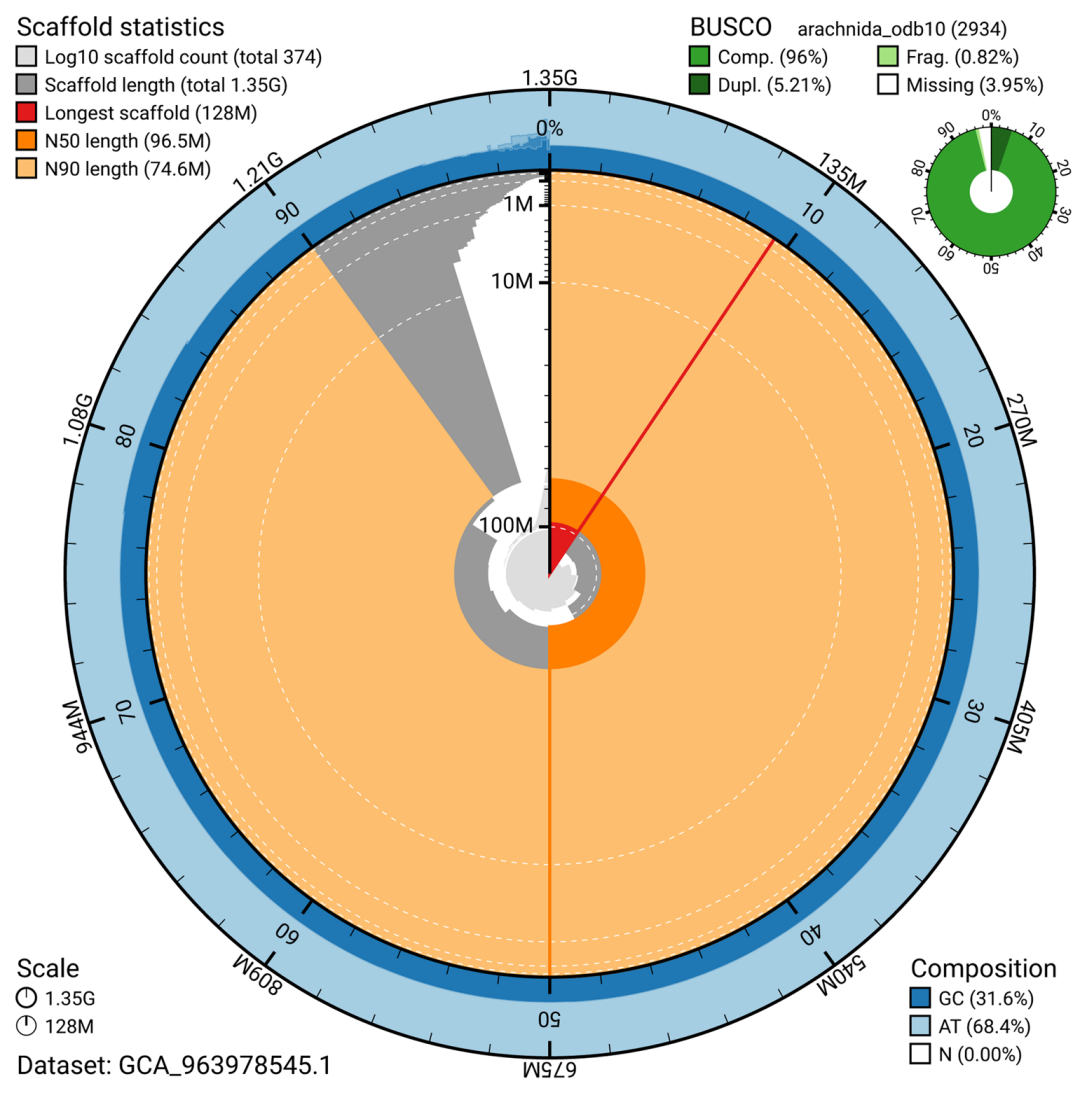
Genome assembly of *Linyphia triangularis*, qqLinTria7.1: metrics. The BlobToolKit snail plot provides an overview of assembly metrics and BUSCO gene completeness. The circumference represents the length of the whole genome sequence, and the main plot is divided into 1,000 bins around the circumference. The outermost blue tracks display the distribution of GC, AT, and N percentages across the bins. Scaffolds are arranged clockwise from longest to shortest and are depicted in dark grey. The longest scaffold is indicated by the red arc, and the deeper orange and pale orange arcs represent the N50 and N90 lengths. A light grey spiral at the centre shows the cumulative scaffold count on a logarithmic scale. A summary of complete, fragmented, duplicated, and missing BUSCO genes in the arachnida_odb10 set is presented at the top right. An interactive version of this figure is available at https://blobtoolkit.genomehubs.org/view/GCA_963978545.1/dataset/GCA_963978545.1/snail.

**Figure 3.**
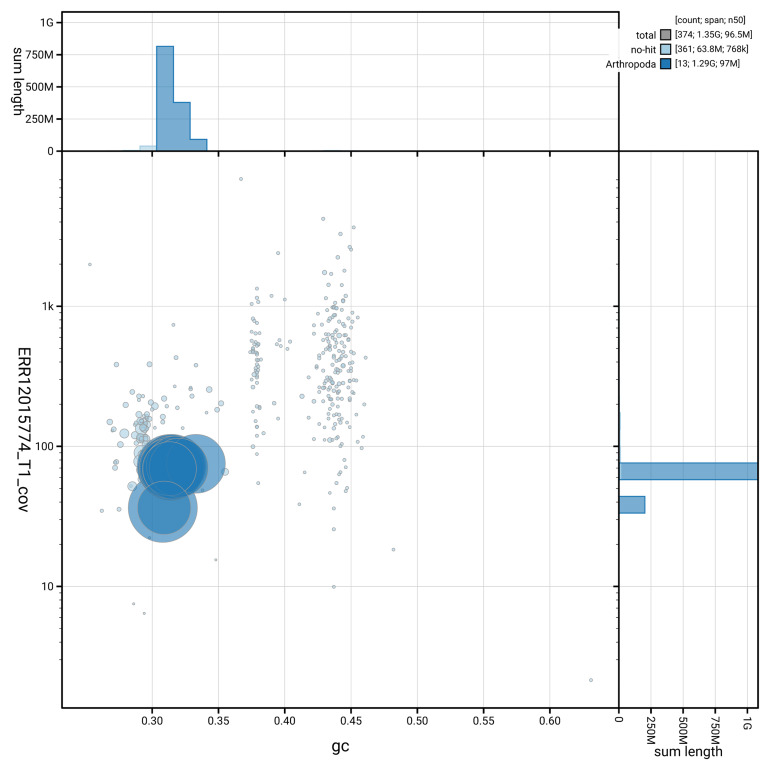
Genome assembly of *Linyphia triangularis*, qqLinTria7.1: BlobToolKit GC-coverage plot. Blob plot showing sequence coverage (vertical axis) and GC content (horizontal axis). The circles represent scaffolds, with the size proportional to scaffold length and the colour representing phylum membership. The histograms along the axes display the total length of sequences distributed across different levels of coverage and GC content. An interactive version of this figure is available at https://blobtoolkit.genomehubs.org/view/GCA_963978545.1/blob.

**Figure 4.**
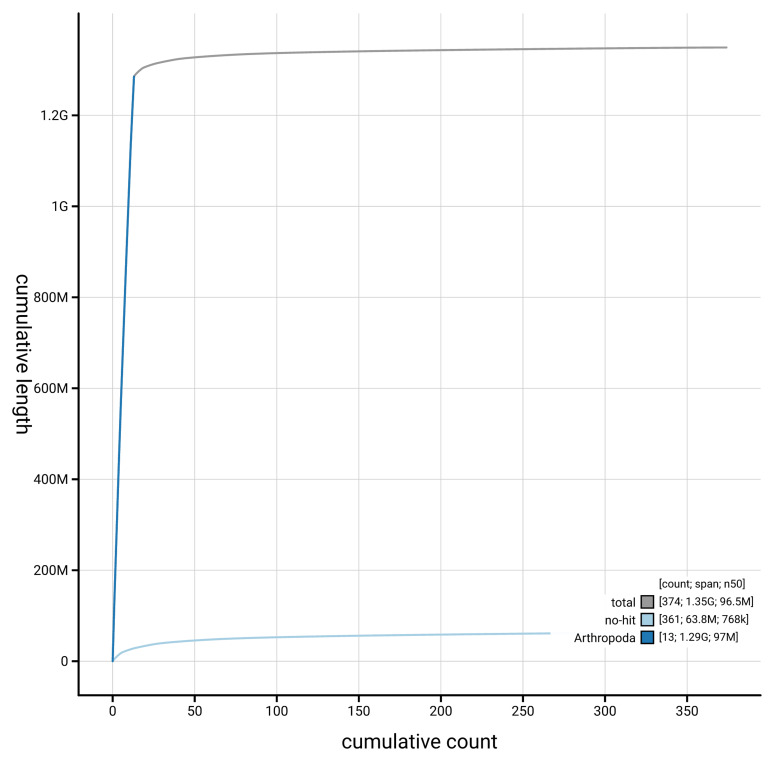
Genome assembly of *Linyphia triangularis,* qqLinTria7.1: BlobToolKit cumulative sequence plot. The grey line shows cumulative length for all scaffolds. Coloured lines show cumulative lengths of scaffolds assigned to each phylum using the buscogenes taxrule. An interactive version of this figure is available at https://blobtoolkit.genomehubs.org/view/GCA_963978545.1/dataset/GCA_963978545.1/cumulative.

The snail plot in Figure 2 provides a summary of the assembly statistics, indicating the distribution of scaffold lengths and other assembly metrics. Figure 3 shows the distribution of scaffolds by GC proportion and coverage. Figure 4 presents a cumulative assembly plot, with separate curves representing different scaffold subsets assigned to various phyla, illustrating the completeness of the assembly.

**Figure 5.**
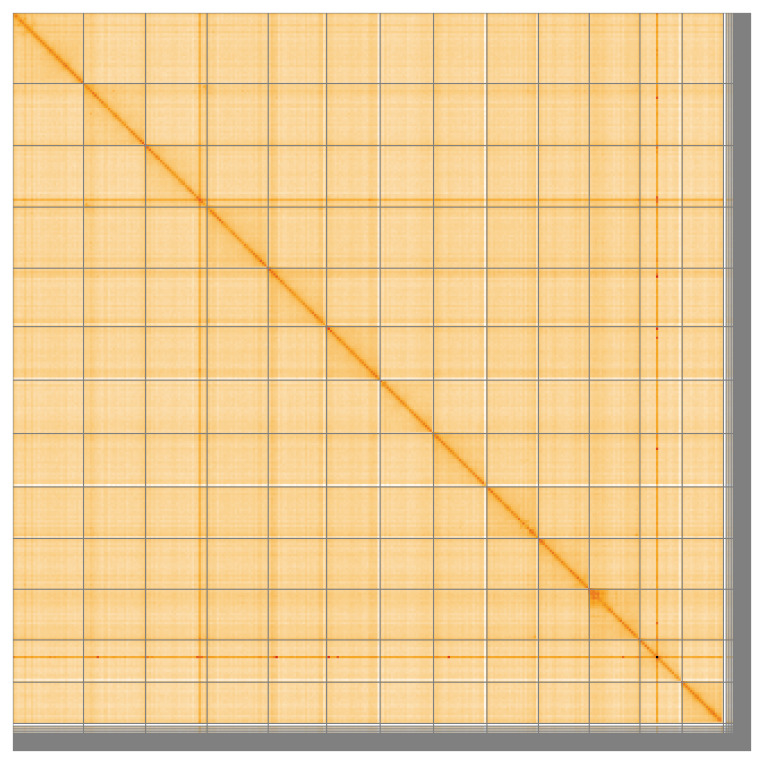
Genome assembly of *Linyphia triangularis:* Hi-C contact map of the qqLinTria7.1 assembly, visualised using HiGlass. Chromosomes are shown in order of size from left to right and top to bottom. An interactive version of this figure may be viewed at https://genome-note-higlass.tol.sanger.ac.uk/l/?d=I9-jz4vWT9WEgiGbqE4g8g.

Most of the assembly sequence (95.28%) was assigned to 13 chromosomal-level scaffolds, representing 11 autosomes and the X _1_ and X _2 _sex chromosome. These chromosome-level scaffolds, confirmed by Hi-C data, are named according to size ( Figure 5; Table 3).

**Table 3.** Chromosomal pseudomolecules in the genome assembly of *Linyphia triangularis*, qqLinTria7.

INSDC accession	Name	Length (Mb)	GC%
OZ021722.1	1	112.14	31.5
OZ021723.1	2	111.4	31.5
OZ021724.1	3	110.54	31.5
OZ021725.1	4	105.52	31.5
OZ021726.1	5	96.96	31.5
OZ021727.1	6	96.46	31.5
OZ021728.1	7	96.45	31.5
OZ021729.1	8	93.26	32
OZ021730.1	9	91.63	33.5
OZ021731.1	10	91.91	32
OZ021732.1	11	76.63	31.5
OZ021734.1	MT	0.02	25.5
OZ021721.1	X1	127.92	31
OZ021733.1	X2	74.56	31

The mitochondrial genome was also assembled. This sequence is included as a contig in the multifasta file of the genome submission and as a standalone record in GenBank.

### Assembly quality metrics

The estimated Quality Value (QV) and *k*-mer completeness metrics, along with BUSCO completeness scores, were calculated for each haplotype and the combined assembly. The QV reflects the base-level accuracy of the assembly, while *k*-mer completeness indicates the proportion of expected *k*-mers identified in the assembly. BUSCO scores provide a measure of completeness based on benchmarking universal single-copy orthologues.

The primary haplotype has a QV of 59.5, and the combined primary and alternate assemblies achieve an estimated QV of 59.6. The *k*-mer completeness for the primary haplotype is 82.97%, and for the alternate haplotype it is 70.52%. The combined primary and alternate assemblies achieve a *k*-mer completeness of 99.10%. BUSCO analysis using the arachnida_odb10 reference set ( *n* = 2,934) indicated a completeness score of 96.0% (single = 90.8%, duplicated = 5.2%).

Table 2 provides assembly metric benchmarks adapted from Rhie *et al.* (2021) and the Earth BioGenome Project Report on Assembly Standards September 2024. The assembly achieves the EBP reference standard of **6.C.Q59**.

## Methods

### Sample acquisition and DNA barcoding

The specimen used for genome sequencing, a male *Linyphia triangularis* (specimen ID NHMUK014042374, ToLID qqLinTria7), was collected from Hartslock Nature Reserve, England, United Kingdom (latitude 51.51, longitude –1.11) on 2021-07-29. The specimen used for Hi-C sequencing (specimen ID NHMUK014452495, ToLID qqLinTria9) was collected on the same occasion. The specimens were collected by Duncan Sivell and Olga Sivell (Natural History Museum), identified by Olga Sivell, and preserved by dry freezing (–80 °C).

The initial identification by morphology was verified by an additional DNA barcoding process according to the framework developed by Twyford *et al*. (2024). A small sample was dissected from the specimen and stored in ethanol, while the remaining parts were shipped on dry ice to the Wellcome Sanger Institute (WSI) ( Pereira *et al.*, 2022). The tissue was lysed, the COI marker region was amplified by PCR, and amplicons were sequenced and compared to the BOLD database, confirming the species identification ( Crowley *et al.*, 2023). Following whole genome sequence generation, the relevant DNA barcode region was also used alongside the initial barcoding data for sample tracking at the WSI ( Twyford *et al.*, 2024). The standard operating procedures for Darwin Tree of Life barcoding have been deposited on protocols.io ( Beasley *et al.*, 2023).

Metadata collection for samples adhered to the Darwin Tree of Life project standards described by Lawniczak *et al*. (2022).

### Nucleic acid extraction

The workflow for high molecular weight (HMW) DNA extraction at the Wellcome Sanger Institute (WSI) Tree of Life Core Laboratory includes a sequence of procedures: sample preparation and homogenisation, DNA extraction, fragmentation and purification. Detailed protocols are available on protocols.io ( Denton *et al.*, 2023b). The qqLinTria7 sample was prepared for DNA extraction by weighing and dissecting it on dry ice ( Jay *et al.*, 2023). Tissue from the head and thorax was homogenised using a PowerMasher II tissue disruptor ( Denton *et al.*, 2023a).

HMW DNA was extracted using the Automated MagAttract v2 protocol ( Oatley *et al.*, 2023a). DNA was sheared into an average fragment size of 12–20 kb in a Megaruptor 3 system ( Bates *et al.*, 2023). Sheared DNA was purified by solid-phase reversible immobilisation, using AMPure PB beads to eliminate shorter fragments and concentrate the DNA ( Oatley *et al.*, 2023b). The concentration of the sheared and purified DNA was assessed using a Nanodrop spectrophotometer and Qubit Fluorometer using the Qubit dsDNA High Sensitivity Assay kit. Fragment size distribution was evaluated by running the sample on the FemtoPulse system.

### Hi-C sample preparation

Tissue from the head and thorax of the qqLinTria9 sample was processed for Hi-C sequencing at the WSI Scientific Operations core, using the Arima-HiC v2 kit. In brief, 20–50 mg of frozen tissue (stored at –80 °C) was fixed, and the DNA crosslinked using a TC buffer with 22% formaldehyde concentration. After crosslinking, the tissue was homogenised using the Diagnocine Power Masher-II and BioMasher-II tubes and pestles. Following the Arima-HiC v2 kit manufacturer's instructions, crosslinked DNA was digested using a restriction enzyme master mix. The 5’-overhangs were filled in and labelled with biotinylated nucleotides and proximally ligated. An overnight incubation was carried out for enzymes to digest remaining proteins and for crosslinks to reverse. A clean up was performed with SPRIselect beads prior to library preparation. Additionally, the biotinylation percentage was estimated using the Qubit Fluorometer v4.0 (Thermo Fisher Scientific) and Qubit HS Assay Kit and Arima-HiC v2 QC beads.

### Library preparation and sequencing

Library preparation and sequencing were performed at the WSI Scientific Operations core.

***PacBio HiFi***

At a minimum, samples were required to have an average fragment size exceeding 8 kb and a total mass over 400 ng to proceed to the low input SMRTbell Prep Kit 3.0 protocol (Pacific Biosciences, California, USA), depending on genome size and sequencing depth required. Libraries were prepared using the SMRTbell Prep Kit 3.0 (Pacific Biosciences, California, USA) as per the manufacturer's instructions. The kit includes the reagents required for end repair/A-tailing, adapter ligation, post-ligation SMRTbell bead cleanup, and nuclease treatment. Following the manufacturer’s instructions, size selection and clean up was carried out using diluted AMPure PB beads (Pacific Biosciences, California, USA). DNA concentration was quantified using the Qubit Fluorometer v4.0 (Thermo Fisher Scientific) with Qubit 1X dsDNA HS assay kit and the final library fragment size analysis was carried out using the Agilent Femto Pulse Automated Pulsed Field CE Instrument (Agilent Technologies) and gDNA 55kb BAC analysis kit.

Samples were sequenced on a Revio instrument (Pacific Biosciences, California, USA). Prepared libraries were normalised to 2 nM, and 15 μL was used for making complexes. Primers were annealed and polymerases were hybridised to create circularised complexes according to manufacturer’s instructions. The complexes were purified with the 1.2X clean up with SMRTbell beads. The purified complexes were then diluted to the Revio loading concentration (in the range 200–300 pM), and spiked with a Revio sequencing internal control. Samples were sequenced on Revio 25M SMRT cells (Pacific Biosciences, California, USA). The SMRT link software, a PacBio web-based end-to-end workflow manager, was used to set-up and monitor the run, as well as perform primary and secondary analysis of the data upon completion.

***Hi-C***

For Hi-C library preparation, DNA was fragmented using the Covaris E220 sonicator (Covaris) and size selected using SPRISelect beads to 400 to 600 bp. The DNA was then enriched using the Arima-HiC v2 kit Enrichment beads. Using the NEBNext Ultra II DNA Library Prep Kit (New England Biolabs) for end repair, a-tailing, and adapter ligation. This uses a custom protocol which resembles the standard NEBNext Ultra II DNA Library Prep protocol but where library preparation occurs while DNA is bound to the Enrichment beads. For library amplification, 10 to 16 PCR cycles were required, determined by the sample biotinylation percentage. The Hi-C sequencing was performed using paired-end sequencing with a read length of 150 bp on an Illumina NovaSeq 6000 instrument.

### Genome assembly, curation and evaluation

***Assembly***

Prior to assembly of the PacBio HiFi reads, a database of *k*-mer counts ( *k* = 31) was generated from the filtered reads using FastK. GenomeScope2 ( Ranallo-Benavidez *et al.*, 2020) was used to analyse the *k*-mer frequency distributions, providing estimates of genome size, heterozygosity, and repeat content.

The HiFi reads were first assembled using Hifiasm ( Cheng *et al.*, 2021) with the --primary option. Haplotypic duplications were identified and removed using purge_dups ( Guan *et al.*, 2020). The Hi-C reads were mapped to the primary contigs using bwa-mem2 ( Vasimuddin *et al.*, 2019). The contigs were further scaffolded using the provided Hi-C data ( Rao *et al.*, 2014) in YaHS ( Zhou *et al.*, 2023) using the --break option for handling potential misassemblies. The scaffolded assemblies were evaluated using Gfastats ( Formenti *et al.*, 2022), BUSCO ( Manni *et al.*, 2021) and MERQURY.FK ( Rhie *et al.*, 2020).

The mitochondrial genome was assembled using MitoHiFi ( Uliano-Silva *et al.*, 2023), which runs MitoFinder ( Allio *et al.*, 2020) and uses these annotations to select the final mitochondrial contig and to ensure the general quality of the sequence.

***Assembly curation***

The assembly was decontaminated using the Assembly Screen for Cobionts and Contaminants (ASCC) pipeline (article in preparation). Flat files and maps used in curation were generated in TreeVal ( Pointon *et al.*, 2023). Manual curation was primarily conducted using PretextView ( Harry, 2022), with additional insights provided by JBrowse2 ( Diesh *et al.*, 2023) and HiGlass ( Kerpedjiev *et al.*, 2018). Scaffolds were visually inspected and corrected as described by Howe *et al*. (2021). Any identified contamination, missed joins, and mis-joins were corrected, and duplicate sequences were tagged and removed. The curation process is documented at https://gitlab.com/wtsi-grit/rapid-curation (article in preparation).

***Assembly quality assessment***

The Merqury.FK tool ( Rhie *et al.*, 2020), run in a Singularity container ( Kurtzer *et al.*, 2017), was used to evaluate *k*-mer completeness and assembly quality for the primary and alternate haplotypes using the *k*-mer databases ( *k* = 31) that were computed prior to genome assembly. The analysis outputs included assembly QV scores and completeness statistics.

A Hi-C contact map was produced for the final version of the assembly. The Hi-C reads were aligned using bwa-mem2 ( Vasimuddin *et al.*, 2019) and the alignment files were combined using SAMtools ( Danecek *et al.*, 2021). The Hi-C alignments were converted into a contact map using BEDTools ( Quinlan & Hall, 2010) and the Cooler tool suite ( Abdennur & Mirny, 2020). The contact map was visualised in HiGlass ( Kerpedjiev *et al.*, 2018).

The blobtoolkit pipeline is a Nextflow port of the previous Snakemake Blobtoolkit pipeline ( Challis *et al.*, 2020). It aligns the PacBio reads in SAMtools and minimap2 ( Li, 2018) and generates coverage tracks for regions of fixed size. In parallel, it queries the GoaT database ( Challis *et al.*, 2023) to identify all matching BUSCO lineages to run BUSCO ( Manni *et al.*, 2021). For the three domain-level BUSCO lineages, the pipeline aligns the BUSCO genes to the UniProt Reference Proteomes database ( Bateman *et al.*, 2023) with DIAMOND blastp ( Buchfink *et al.*, 2021). The genome is also divided into chunks according to the density of the BUSCO genes from the closest taxonomic lineage, and each chunk is aligned to the UniProt Reference Proteomes database using DIAMOND blastx. Genome sequences without a hit are chunked using seqtk and aligned to the NT database with blastn ( Altschul *et al.*, 1990). The blobtools suite combines all these outputs into a blobdir for visualisation.

The blobtoolkit pipeline was developed using nf-core tooling ( Ewels *et al.*, 2020) and MultiQC ( Ewels *et al.*, 2016), relying on the Conda package manager, the Bioconda initiative ( Grüning *et al.*, 2018), the Biocontainers infrastructure ( da Veiga Leprevost *et al.*, 2017), as well as the Docker ( Merkel, 2014) and Singularity ( Kurtzer *et al.*, 2017) containerisation solutions.

Table 4 contains a list of relevant software tool versions and sources.

**Table 4.** Software tools: versions and sources.

Software tool	Version	Source
BEDTools	2.30.0	https://github.com/arq5x/bedtools2
BLAST	2.14.0	ftp://ftp.ncbi.nlm.nih.gov/blast/executables/blast+/
BlobToolKit	4.3.9	https://github.com/blobtoolkit/blobtoolkit
BUSCO	5.5.0	https://gitlab.com/ezlab/busco
bwa-mem2	2.2.1	https://github.com/bwa-mem2/bwa-mem2
Cooler	0.8.11	https://github.com/open2c/cooler
DIAMOND	2.1.8	https://github.com/bbuchfink/diamond
fasta_ windows	0.2.4	https://github.com/tolkit/fasta_windows
FastK	427104ea91c78c3b8b8b49f1a7d6bbeaa869ba1c	https://github.com/thegenemyers/FASTK
Gfastats	1.3.6	https://github.com/vgl-hub/gfastats
GoaT CLI	0.2.5	https://github.com/genomehubs/goat-cli
Hifiasm	0.19.5-r587	https://github.com/chhylp123/hifiasm
HiGlass	44086069ee7d4d3f6f3f0012569789ec138f42b84a a44357826c0b6753eb28de	https://github.com/higlass/higlass
MerquryFK	d00d98157618f4e8d1a9190026b19b471055b22e	https://github.com/thegenemyers/MERQURY.FK
Minimap2	2.24-r1122	https://github.com/lh3/minimap2
MitoHiFi	3	https://github.com/marcelauliano/MitoHiFi
MultiQC	1.14, 1.17, and 1.18	https://github.com/MultiQC/MultiQC
NCBI Datasets	15.12.0	https://github.com/ncbi/datasets
Nextflow	23.04.1	https://github.com/nextflow-io/nextflow
PretextView	0.2.5	https://github.com/sanger-tol/PretextView
purge_dups	1.2.5	https://github.com/dfguan/purge_dups
samtools	1.19.2	https://github.com/samtools/samtools
sanger-tol/ ascc	-	https://github.com/sanger-tol/ascc
sanger-tol/ blobtoolkit	0.5.1	https://github.com/sanger-tol/blobtoolkit
Seqtk	1.3	https://github.com/lh3/seqtk
Singularity	3.9.0	https://github.com/sylabs/singularity
TreeVal	1.2.0	https://github.com/sanger-tol/treeval
YaHS	1.2a.2	https://github.com/c-zhou/yahs

### Wellcome Sanger Institute – Legal and Governance

The materials that have contributed to this genome note have been supplied by a Darwin Tree of Life Partner. The submission of materials by a Darwin Tree of Life Partner is subject to the **‘Darwin Tree of Life Project Sampling Code of Practice’**, which can be found in full on the Darwin Tree of Life website here. By agreeing with and signing up to the Sampling Code of Practice, the Darwin Tree of Life Partner agrees they will meet the legal and ethical requirements and standards set out within this document in respect of all samples acquired for, and supplied to, the Darwin Tree of Life Project.

Further, the Wellcome Sanger Institute employs a process whereby due diligence is carried out proportionate to the nature of the materials themselves, and the circumstances under which they have been/are to be collected and provided for use. The purpose of this is to address and mitigate any potential legal and/or ethical implications of receipt and use of the materials as part of the research project, and to ensure that in doing so we align with best practice wherever possible. The overarching areas of consideration are:

•   Ethical review of provenance and sourcing of the material

•   Legality of collection, transfer and use (national and international) 

Each transfer of samples is further undertaken according to a Research Collaboration Agreement or Material Transfer Agreement entered into by the Darwin Tree of Life Partner, Genome Research Limited (operating as the Wellcome Sanger Institute), and in some circumstances other Darwin Tree of Life collaborators.

## Data Availability

European Nucleotide Archive: Linyphia triangularis (European sheetweb spider). Accession number PRJEB65733; https://identifiers.org/ena.embl/PRJEB65733. The genome sequence is released openly for reuse. The *Linyphia triangularis* genome sequencing initiative is part of the Darwin Tree of Life (DToL) project. All raw sequence data and the assembly have been deposited in INSDC databases. The genome will be annotated using available RNA-Seq data and presented through the Ensembl pipeline at the European Bioinformatics Institute. Raw data and assembly accession identifiers are reported in Table 1 and Table 2.

## Author information

Members of the Natural History Museum Genome Acquisition Lab are listed here: https://doi.org/10.5281/zenodo.12159242.

Members of the Darwin Tree of Life Barcoding collective are listed here: https://doi.org/10.5281/zenodo.12158331.

Members of the Wellcome Sanger Institute Tree of Life Management, Samples and Laboratory team are listed here: https://doi.org/10.5281/zenodo.12162482.

Members of Wellcome Sanger Institute Scientific Operations: Sequencing Operations are listed here: https://doi.org/10.5281/zenodo.12165051.

Members of the Wellcome Sanger Institute Tree of Life Core Informatics team are listed here: https://doi.org/10.5281/zenodo.12160324.

Members of the Tree of Life Core Informatics collective are listed here: https://doi.org/10.5281/zenodo.12205391.

Members of the Darwin Tree of Life Consortium are listed here: https://doi.org/10.5281/zenodo.4783558.

## References

[ref-1] Abdennur N Mirny LA : Cooler: scalable storage for Hi-C data and other genomically labeled arrays. *Bioinformatics.* 2020;36(1):311–316. 10.1093/bioinformatics/btz540 31290943 PMC8205516

[ref-2] Allio R Schomaker-Bastos A Romiguier J : MitoFinder: efficient automated large-scale extraction of mitogenomic data in target enrichment phylogenomics. *Mol Ecol Resour.* 2020;20(4):892–905. 10.1111/1755-0998.13160 32243090 PMC7497042

[ref-3] Altschul SF Gish W Miller W : Basic local alignment search tool. *J Mol Biol.* 1990;215(3):403–410. 10.1016/S0022-2836(05)80360-2 2231712

[ref-66] Arnedo MA Hormiga G Scharff N : Higher‐level phylogenetics of linyphiid spiders (Araneae, *Linyphiidae*) based on morphological and molecular evidence. *Cladistics.* 2009;25(3):231–262. 10.1111/j.1096-0031.2009.00249.x 34879614

[ref-4] Bateman A Martin MJ Orchard S : UniProt: the universal protein knowledgebase in 2023. *Nucleic Acids Res.* 2023;51(D1):D523–D531. 10.1093/nar/gkac1052 36408920 PMC9825514

[ref-5] Bates A Clayton-Lucey I Howard C : Sanger Tree of Life HMW DNA fragmentation: diagenode Megaruptor ^®^3 for LI PacBio. *protocols.io.* 2023. 10.17504/protocols.io.81wgbxzq3lpk/v1

[ref-6] Beasley J Uhl R Forrest LL : DNA barcoding SOPs for the Darwin Tree of Life project. *protocols.io.* 2023; [Accessed 25 June 2024]. 10.17504/protocols.io.261ged91jv47/v1

[ref-67] Bednarski J Ginsberg H Jakob EM : Competitive interactions between a native spider ( *Frontinella communis,* Araneae: *Linyphiidae*) and an invasive spider ( *Linyphia triangularis,* Araneae: *Linyphiidae*). *Biol Invasions.* 2010;12:905–912. 10.1007/s10530-009-9511-7

[ref-68] Bee L Oxford G Smith H : Britain’s spiders: a field guide.Princeton University Press,2017. Reference Source

[ref-69] Benjamin SP Düggelin M Zschokke S : Fine structure of sheet‐webs of *Linyphia triangularis* (Clerck) and *Microlinyphia pusilla* (Sundevall), with remarks on the presence of viscid silk. *Acta Zool.* 2002;83(1):49–59. 10.1046/j.1463-6395.2002.00098.x

[ref-70] Benjamin SP Zschokke S : Homology, behaviour and spider webs: web construction behaviour of *Linyphia hortensis* and *L. triangularis* (Araneae: *Linyphiidae*) and its evolutionary significance. *J Evol Biol.* 2004;17(1):120–130. 10.1046/j.1420-9101.2004.00667.x 15000655

[ref-7] Buchfink B Reuter K Drost HG : Sensitive protein alignments at Tree-of-Life scale using DIAMOND. *Nat Methods.* 2021;18(4):366–368. 10.1038/s41592-021-01101-x 33828273 PMC8026399

[ref-8] Challis R Kumar S Sotero-Caio C : Genomes on a Tree (GoaT): a versatile, scalable search engine for genomic and sequencing project metadata across the eukaryotic Tree of Life [version 1; peer review: 2 approved]. *Wellcome Open Res.* 2023;8:24. 10.12688/wellcomeopenres.18658.1 36864925 PMC9971660

[ref-9] Challis R Richards E Rajan J : BlobToolKit – interactive quality assessment of genome assemblies. *G3 (Bethesda).* 2020;10(4):1361–1374. 10.1534/g3.119.400908 32071071 PMC7144090

[ref-10] Cheng H Concepcion GT Feng X : Haplotype-resolved *de novo* assembly using phased assembly graphs with hifiasm. *Nat Methods.* 2021;18(2):170–175. 10.1038/s41592-020-01056-5 33526886 PMC7961889

[ref-11] Crowley L Allen H Barnes I : A sampling strategy for genome sequencing the British terrestrial arthropod fauna [version 1; peer review: 2 approved]. *Wellcome Open Res.* 2023;8:123. 10.12688/wellcomeopenres.18925.1 37408610 PMC10318377

[ref-12] da Veiga Leprevost F Grüning BA Alves Aflitos S : BioContainers: an open-source and community-driven framework for software standardization. *Bioinformatics.* 2017;33(16):2580–2582. 10.1093/bioinformatics/btx192 28379341 PMC5870671

[ref-13] Danecek P Bonfield JK Liddle J : Twelve years of SAMtools and BCFtools. *GigaScience.* 2021;10(2): giab008. 10.1093/gigascience/giab008 33590861 PMC7931819

[ref-14] Denton A Oatley G Cornwell C : Sanger Tree of Life sample homogenisation: PowerMash. *protocols.io.* 2023a. 10.17504/protocols.io.5qpvo3r19v4o/v1

[ref-15] Denton A Yatsenko H Jay J : Sanger Tree of Life wet laboratory protocol collection V.1. *protocols.io.* 2023b. 10.17504/protocols.io.8epv5xxy6g1b/v1

[ref-44] Diesh C Stevens GJ Xie P : JBrowse 2: a modular genome browser with views of synteny and structural variation. *Genome Biol.* 2023;24(1): 74. 10.1186/s13059-023-02914-z 37069644 PMC10108523

[ref-18] Ewels P Magnusson M Lundin S : MultiQC: summarize analysis results for multiple tools and samples in a single report. *Bioinformatics.* 2016;32(19):3047–3048. 10.1093/bioinformatics/btw354 27312411 PMC5039924

[ref-17] Ewels PA Peltzer A Fillinger S : The nf-core framework for community-curated bioinformatics pipelines. *Nat Biotechnol.* 2020;38(3):276–278. 10.1038/s41587-020-0439-x 32055031

[ref-19] Formenti G Abueg L Brajuka A : Gfastats: conversion, evaluation and manipulation of genome sequences using assembly graphs. *Bioinformatics.* 2022;38(17):4214–4216. 10.1093/bioinformatics/btac460 35799367 PMC9438950

[ref-71] Gavish-Regev E Hormiga G Scharff N : Pedipalp sclerite homologies and phylogenetic placement of the spider genus *Stemonyphantes* (Linyphiidae, Araneae) and its implications for linyphiid phylogeny. *Invertebrate Systematics.* 2013;27(1):38–52. 10.1071/IS12014

[ref-24] GBIF Secretariat : *Linyphia triangularis* (Clerck, 1757) map on GBIF. 2024. Reference Source

[ref-20] Grüning B Dale R Sjödin A : Bioconda: sustainable and comprehensive software distribution for the life sciences. *Nat Methods.* 2018;15(7):475–476. 10.1038/s41592-018-0046-7 29967506 PMC11070151

[ref-21] Guan D McCarthy SA Wood J : Identifying and removing haplotypic duplication in primary genome assemblies. *Bioinformatics.* 2020;36(9):2896–2898. 10.1093/bioinformatics/btaa025 31971576 PMC7203741

[ref-22] Harry E : PretextView (Paired REad TEXTure Viewer): a desktop application for viewing pretext contact maps. 2022. Reference Source

[ref-72] Harvey PR Nellist DR Telfer MG : Provisional atlas of British spiders (Arachnida, Araneae).Biological Records Centre, Centre for Ecology and Hydrology,2002;1. Reference Source

[ref-73] Hormiga G : Higher level phylogenetics of erigonine spiders ( *Araneae,* Linyphiidae, Erigoninae). *Smithson Contrib Zool.* 2000;609:1–160. 10.5479/si.00810282.609

[ref-23] Hormiga G Scharff N : Monophyly and phylogenetic placement of the spider genus *Labulla* Simon, 1884 ( *Araneae*, Linyphiidae) and description of the new genus *Pecado*. *Zool J Linn Soc.* 2005;143:359–404. Reference Source

[ref-26] Houser JD Ginsberg H Jakob EM : Competition between introduced and native spiders ( *Araneae*: Linyphiidae). *Biol Invasions.* 2014;16:2479–2488. 10.1007/s10530-014-0679-0

[ref-27] Howe K Chow W Collins J : Significantly improving the quality of genome assemblies through curation. *GigaScience.* 2021;10(1): giaa153. 10.1093/gigascience/giaa153 33420778 PMC7794651

[ref-28] Jay J Yatsenko H Narváez-Gómez JP : Sanger Tree of Life sample preparation: triage and dissection. *protocols.io.* 2023. 10.17504/protocols.io.x54v9prmqg3e/v1

[ref-74] Jennings DT Catley KM Graham F Jr : *Linyphia triangularis,* a palearctic spider (Araneae, Linyphiidae) new to North America. *J Arachnol.* 2002;30(3):455–460. Reference Source

[ref-25] Kerpedjiev P Abdennur N Lekschas F : HiGlass: web-based visual exploration and analysis of genome interaction maps. *Genome Biol.* 2018;19(1): 125. 10.1186/s13059-018-1486-1 30143029 PMC6109259

[ref-29] Kurtzer GM Sochat V Bauer MW : Singularity: scientific containers for mobility of compute. *PLoS One.* 2017;12(5): e0177459. 10.1371/journal.pone.0177459 28494014 PMC5426675

[ref-30] Lawniczak MKN Davey RP Rajan J : Specimen and sample metadata standards for biodiversity genomics: a proposal from the Darwin Tree of Life project [version 1; peer review: 2 approved with reservations]. *Wellcome Open Res.* 2022;7:187. 10.12688/wellcomeopenres.17605.1

[ref-31] Li H : Minimap2: pairwise alignment for nucleotide sequences. *Bioinformatics.* 2018;34(18):3094–3100. 10.1093/bioinformatics/bty191 29750242 PMC6137996

[ref-32] Manni M Berkeley MR Seppey M : BUSCO update: novel and streamlined workflows along with broader and deeper phylogenetic coverage for scoring of eukaryotic, prokaryotic, and viral genomes. *Mol Biol Evol.* 2021;38(10):4647–4654. 10.1093/molbev/msab199 34320186 PMC8476166

[ref-33] Merkel D : Docker: lightweight Linux containers for consistent development and deployment. *Linux J.* 2014;2014(239): 2, [Accessed 2 April 2024]. Reference Source

[ref-75] Miller JA Hormiga G : Clade stability and the addition of data: a case study from erigonine spiders ( *Araneae*: Linyphiidae, Erigoninae). *Cladistics.* 2004;20(5):385–442. 10.1111/j.1096-0031.2004.00033.x 34892954

[ref-76] Nielsen N Toft S : Alternative male mating strategies in *Linyphia triangularis* ( *Araneae,* Linyphiidae). *Acta Zoologica Fennica.* 1990;190:293–297. Reference Source

[ref-34] Oatley G Denton A Howard C : Sanger Tree of Life HMW DNA extraction: automated MagAttract v.2. *protocols.io.* 2023a. 10.17504/protocols.io.kxygx3y4dg8j/v1

[ref-35] Oatley G Sampaio F Howard C : Sanger Tree of Life fragmented DNA clean up: automated SPRI. *protocols.io.* 2023b. 10.17504/protocols.io.q26g7p1wkgwz/v1

[ref-36] Pereira L Sivell O Sivess L : DToL Taxon-specific standard operating Procedure for the terrestrial and freshwater arthropods working group. 2022. 10.17504/protocols.io.261gennyog47/v1

[ref-37] Pointon DL Eagles W Sims Y : sanger-tol/treeval v1.0.0 – Ancient Atlantis. 2023. 10.5281/zenodo.10047654

[ref-38] Quinlan AR Hall IM : BEDTools: a flexible suite of utilities for comparing genomic features. *Bioinformatics.* 2010;26(6):841–842. 10.1093/bioinformatics/btq033 20110278 PMC2832824

[ref-39] Ranallo-Benavidez TR Jaron KS Schatz MC : GenomeScope 2.0 and Smudgeplot for reference-free profiling of polyploid genomes. *Nat Commun.* 2020;11(1): 1432. 10.1038/s41467-020-14998-3 32188846 PMC7080791

[ref-40] Rao SSP Huntley MH Durand NC : A 3D map of the human genome at kilobase resolution reveals principles of chromatin looping. *Cell.* 2014;159(7):1665–1680. 10.1016/j.cell.2014.11.021 25497547 PMC5635824

[ref-52] Rhie A McCarthy SA Fedrigo O : Towards complete and error-free genome assemblies of all vertebrate species. *Nature.* 2021;592(7856):737–746. 10.1038/s41586-021-03451-0 33911273 PMC8081667

[ref-53] Rhie A Walenz BP Koren S : Merqury: reference-free quality, completeness, and phasing assessment for genome assemblies. *Genome Biol.* 2020;21(1): 245. 10.1186/s13059-020-02134-9 32928274 PMC7488777

[ref-54] Roberts MJ : Collins field guide. Spiders of Britain and northern Europe.HarperCollins Publishers Ltd,1996.

[ref-55] Rovner JS : Territoriality in the sheet-web spider *Linyphia triangularis* (Araneae, Linyphiidae). *Z Tierpsychol.* 1968;25(2):232–42. 10.1111/j.1439-0310.1968.tb00015.x 5693334

[ref-56] Schulz S Toft S : Identification of a sex pheromone from a spider. *Science.* 1993;260(5114):1635–7. 10.1126/science.260.5114.1635 17810206

[ref-57] Simpson S Sellers E Pagad S : Global Register of Introduced and Invasive Species - United States (Contiguous) (ver.2.0, 2022). *Invasive Species Specialist Group (ISSG).* 2023. 10.5066/p9kfftod

[ref-77] Stumpf H : Observations on the copulation behaviour of the sheet-web spiders *Linyphia hortensis* Sundevall and *Linyphia triangularis* (Clerck) ( *Araneae*: Linyphiidae). *Bulletin of the Society of European Arachnology.* 1990;1:340–345.

[ref-78] Toft S : Mate guarding in two *Linyphia* species (Araneae: Linyphiidae). *Bull Br Arachnol Soc.* 1989;8(2):33–37. Reference Source

[ref-79] Tu L Hormiga G : Phylogenetic analysis and revision of the linyphiid spider genus *Solenysa* ( *Araneae*: Linyphiidae: Erigoninae). *Zool J Linn Soc.* 2011;161(3):484–530. 10.1111/j.1096-3642.2010.00640.x

[ref-58] Twyford AD Beasley J Barnes I : A DNA barcoding framework for taxonomic verification in the Darwin Tree of Life project [version 1; peer review: 2 approved]. *Wellcome Open Res.* 2024;9:339. 10.12688/wellcomeopenres.21143.1 39386966 PMC11462125

[ref-41] Uliano-Silva M Ferreira JGRN Krasheninnikova K : MitoHiFi: a python pipeline for mitochondrial genome assembly from PacBio high fidelity reads. *BMC Bioinformatics.* 2023;24(1): 288. 10.1186/s12859-023-05385-y 37464285 PMC10354987

[ref-42] Vasimuddin M Misra S Li H : Efficient architecture-aware acceleration of BWA-MEM for multicore systems.In: *2019 IEEE International Parallel and Distributed Processing Symposium (IPDPS).*IEEE,2019;314–324. 10.1109/IPDPS.2019.00041

[ref-80] Wang F Ballesteros JA Hormiga G : Resolving the phylogeny of a speciose spider group, the family Linyphiidae (Araneae). *Mol Phylogenet Evol.* 2015;91:135–149. 10.1016/j.ympev.2015.05.005 25988404

[ref-81] Weldingh DL Toft S Larsen ON : Mating duration and sperm precedence in the spider *Linyphia triangularis.* *J Ethol.* 2011;29:143–152. 10.1007/s10164-010-0237-x

[ref-43] Zhou C McCarthy SA Durbin R : YaHS: yet another Hi-C scaffolding tool. *Bioinformatics.* 2023;39(1): btac808. 10.1093/bioinformatics/btac808 36525368 PMC9848053

