# Supplementary material for: The genome sequence of the Common Sheetweb Spider Linyphia triangularis (Clerck, 1757)
Source: Wellcome Open Res. 2025 Feb 24;10:92. [Version 1] doi: 10.12688/wellcomeopenres.23754.1 (PMC11904403; doi:10.12688/wellcomeopenres.23754.1)
Supplement: Peer review history

# Reviewer report: Reviewer response for version 1

**DOI:** 10.21956/wellcomeopenres.26202.r119455

**Published:** 2025-03-12

**Authors:** Miquel Arnedo

**Affiliations:** 1 Universitat de Barcelona, Barcelona, Spain

Overall, I think that the information provided is both complete and accurate. I only have some minor concerns regarding a few references.

 For example, when referring to references for species identification, it is better to refer to https://araneae.nmbe.ch/data/1256, which includes drawings for both sexes, a dichotomous key, and more detailed information.

 Additionally, the paragraph about the phylogenetic position, including the monophyly and boundaries of Linyphiidae, is a bit outdated. These two aspects have been more recently investigated using both transcriptomics and UCEs. An up-to-date summary of the current state of affairs can be found here: Kulkarni, S., Wood, H. M., & Hormiga, G. (2023). Advances in the reconstruction of the spider treemap for spider systematics and comparative study of life: A roadmap. Cladistics, 39, 479-532. https://doi.org/10.1111/cla.12557

 Finally, I have not found any part that mentions the sexes or stages of the individuals used for the analyses. This is particularly relevant since sex determination in the group is chromosomal: X1X20 for males.

Are sufficient details of methods and materials provided to allow replication by others?

Partly

Is the rationale for creating the dataset(s) clearly described?

Yes

Are the datasets clearly presented in a useable and accessible format?

Yes

Are the protocols appropriate and is the work technically sound?

Yes

Reviewer Expertise:

Systematic biology, evolutionary biology

I confirm that I have read this submission and believe that I have an appropriate level of expertise to confirm that it is of an acceptable scientific standard.

## References

[1] : Advances in the reconstruction of the spider tree of life: A roadmap for spider systematics and comparative studies. *Cladistics* .2023;39(6) : 10.1111/cla.12557 479-532 10.1111/cla.12557 37787157

[2] Spiders of Europe. Version {no. of month}.2025. araneae Version 03.2025 Nentwig W, Blick T, Bosmans R Hänggi A, Kropf C, Stäubli A (2025). *accessed on {date of access}.* . Reference source

[3] *araneae Spider of Europe* . Reference source

# Reviewer report: Reviewer response for version 1

**DOI:** 10.21956/wellcomeopenres.26202.r119452

**Published:** 2025-03-05

**Authors:** Madeleine E Aase-Remedios

**Affiliations:** 1 Durham University, Durham, England, UK

The authors present a high quality genome assembly for the common sheetweb spider *Linyphia triangularis* for public use, using state of the art methods. Rationale is explicitly given in resolving the Linyphiidae phylogeny, and as part of the larger DToL project's sequencing goal.

 No RNA was sequenced and the genome is unannotated, which is a drawback to the usefulness of this resource.

 The methods include details to make this replicable (programs and parameters used, etc.), among the links to the standard pipeline, protocols, and accessions.

 The background provides an extensive summary of the identifying marks, habitat, and behaviour of the species, including its invasion into North America. Overall, the content is more than sufficient for a data note.

 Typographical:

 - Insert "the" between "after" and "female's" in "...and copulation occurs immediately after female’s final moult"

 - Remove "The" from "The further work is needed to resolve phylogenetic relationships within Linyphyiidae"

Are sufficient details of methods and materials provided to allow replication by others?

Yes

Is the rationale for creating the dataset(s) clearly described?

Yes

Are the datasets clearly presented in a useable and accessible format?

Yes

Are the protocols appropriate and is the work technically sound?

Yes

Reviewer Expertise:

Spider genomics, evolution, development

I confirm that I have read this submission and believe that I have an appropriate level of expertise to confirm that it is of an acceptable scientific standard.
